# Unique secreted–surface protein complex of *Lactobacillus rhamnosus*, identified by phage display

**DOI:** 10.1002/mbo3.53

**Published:** 2012-12-11

**Authors:** Dragana Gagic, Wesley Wen, Michael A Collett, Jasna Rakonjac

**Affiliations:** 1 Institute of Molecular BioSciences, Massey UniversityPrivate Bag 11222, Palmerston North 4442, New Zealand; 2 Fonterra Research and Development CentrePrivate Bag 11029, Palmerston North 4442, New Zealand

**Keywords:** Adhesins, bacteriophages, cell surface, *Lactobacillus*

## Abstract

Proteins are the most diverse structures on bacterial surfaces; hence, they are candidates for species- and strain-specific interactions of bacteria with the host, environment, and other microorganisms. Genomics has decoded thousands of bacterial surface and secreted proteins, yet the function of most cannot be predicted because of the enormous variability and a lack of experimental data that would allow deduction of function through homology. Here, we used phage display to identify a pair of interacting extracellular proteins in the probiotic bacterium *Lactobacillus rhamnosus* HN001. A secreted protein, SpcA, containing two bacterial immunoglobulin-like domains type 3 (Big-3) and a domain distantly related to plant pathogen response domain 1 (PR-1-like) was identified by screening of an *L. rhamnosus* HN001 library using HN001 cells as bait. The SpcA-“docking” protein, SpcB, was in turn detected by another phage display library screening, using purified SpcA as bait. SpcB is a 3275-residue cell-surface protein that contains general features of large glycosylated Serine-rich adhesins/fibrils from gram-positive bacteria, including the hallmark signal sequence motif KxYKxGKxW. Both proteins are encoded by genes within a *L. rhamnosus*-unique gene cluster that distinguishes this species from other lactobacilli. To our knowledge, this is the first example of a secreted-docking protein pair identified in lactobacilli.

## Introduction

Bacterial surface proteins have been studied extensively in pathogenic bacteria over the last several decades, demonstrating their role in host colonization and cell invasion as well as very sophisticated manipulation of the host immune response pathways that often neutralize microbial-pattern-induced signaling pathways (Patti et al. 1994; Beckmann et al. 2002; Selbach and Backert 2005; Lilic et al. 2006; Luck et al. 2006; Timmer et al. 2006; Eskan et al. 2007; Gillen et al. 2008; Kulkarni et al. 2009; McGhie et al. 2009). Together with the diversity of nonprotein surface molecules and structures (cell wall peptidoglycan, lipotheicoic acid, and exopolysaccharides), surface and secreted proteins determine the overall interaction of a pathogen with the host and the outcomes of the pathogen colonization (Grandel and Grimminger 2003; Boltana et al. 2011; Elberse et al. 2011; Hao et al. 2011; Tuanyok et al. 2012). Complex interactions with the host, including colonization and manipulation of immune responses, are not limited to pathogens. Commensal bacteria that colonize the gastrointestinal tract were found to be engaged in complex bacteria–host and bacteria–bacteria interactions that are mediated in part by surface and secreted proteins (Kolenbrander et al. 2006; Sansonetti and Medzhitov 2009; Sansonetti 2011). The advent of metagenomics brought appreciation for the diversity of microbial communities inhabiting the gastrointestinal system and other surfaces that are exposed to the environment (Grice and Segre 2011; Jenkinson 2011; Walter and Ley 2011; Dave et al. 2012), and understanding that the human gut–microbe interactions are in a state of immunological homeostasis in healthy individuals (Rimoldi et al. 2005; Sansonetti and Medzhitov 2009; Kinross et al. 2011). This homeostasis is disrupted when pathogens invade or expand, causing damage to the gut lining and profound inflammation (Sansonetti 2004). To restore the balance, preparations of live beneficial bacteria, called probiotics, are often recommended. Information on their role in the recovery from gut damage by pathogens and inflammation comes from animal models and clinical studies (Konstantinov et al. 2008; Preidis et al. 2011; Swanson et al. 2011; Wells 2011; Bron et al. 2012; Hummel et al. 2012). However, the surface or secreted proteins of the probiotic bacteria that likely mediate these beneficial effects are scarcely known (van Pijkeren et al. 2006; Kleerebezem et al. 2010; Wells 2011; Bron et al. 2012).

*Lactobacillus rhamnosus* is a species from the genus *Lactobacillus*, represented by several strains with demonstrated probiotic effects. The most prominent probiotic strains of *L. rhamnosus* are GG and HN001 (Gill et al. 2001; Sheih et al. 2001; Cross et al. 2002; Shu and Gill 2002; Lin et al. 2009; Thomas et al. 2011; Lahtinen et al. 2012). These two strains demonstrate aggregation in culture (Collado et al. 2007; Dekker et al. 2007). Both strains have been reported to have similar beneficial effects, including protection from enteric pathogens and prevention of eczema (Gill et al. 2001; Kalliomaki et al. 2003; Collado et al. 2006; Wickens et al. 2012).

A number of other lactobacilli species have been characterized as probiotics; some of those are isolates from dairy fermentation, whereas others are commensals (i.e., members of the resident gut microbial communities) (Wells 2011). Genome sequencing of a number of these species and strains have revealed a large number of diverse surface and secreted proteins, many of which are thought to have similar colonization roles as observed in bacterial pathogens (van Pijkeren et al. 2006; Jankovic et al. 2007; Kleerebezem et al. 2010), such as mucin- and fibronectin-binding proteins (Toba et al. 1995; Altermann et al. 2005; Buck et al. 2005; Castaldo et al. 2009; Munoz-Provencio et al. 2010). Genomic information for a large number of poorly characterized commensal lactobacilli species is being collected within the United States and European human microbial genome projects (Dave et al. 2012).

Despite a large amount of genomic data on probiotic and commensal organisms, the functional understanding of extracellular (surface and secreted) proteins is far behind the model pathogenic bacteria (van Pijkeren et al. 2006; Kowalchuk et al. 2007; Song et al. 2009; Kleerebezem et al. 2010; Zhou et al., 2010; Kinross et al. 2011; Walter and Ley 2011). The high diversity of extracellular proteins means that their function, even in pathogenic bacteria, is not possible to identify by bioinformatics, in particular because the sequence similarity between proteins that have the same function is often limited to a genus, species, and even strain (e.g., proteins implicated in binding to the mucosal surfaces, or manipulation of immune response). These proteins therefore appear to represent recent evolutionary adaptations (Baltrus et al. 2011; Carroll et al. 2011).

One approach in identifying proteins that carry out a particular function, which cannot be derived from bioinformatics analyses, is to identify them by this function. This usually involves screening of expression libraries for activity or interactions. In this realm, a combinatorial affinity-selection method, called phage display (Zwick et al., 1998; Forrer et al. 1999; Sidhu et al. 2003; Rakonjac et al. 2011), has been successfully used to find extracellular proteins in pathogenic bacteria that interact with complex targets, such as bacterial or host cells, extracellular matrix proteins, or host antibody repertoire (Mullen et al. 2006). Phage display has also been used recently to identify the fibronectin-binding proteins from commensal bacterium *Lactobacillus casei* BL23 (Munoz-Provencio and Monedero 2011).

Cell-binding proteins of bacteria are often enzymes or proteins that mediate aggregation or biofilm formation. We used phage display to identify a cell-binding protein that interacts with the surface of HN001 cells (which we named SpcA), and its cognate “docking” cell-surface protein (named SpcB). The secreted protein SpcA has two bacterial immunoglobulin-like domains type 3 (Big-3) that mediate binding to the docking protein SpcB and a C-terminal domain of unknown function that is distantly related to plant pathogen response protein 1 (PR-1). The docking protein SpcB is the largest protein in *L. rhamnosus* genome; it has a standard LPXTG cell wall anchor motif, whereas its signal sequence and overall organization are characteristic of gigantic glycosylated Ser-rich proteins. Both proteins are unique to *L. rhamnosus*.

## Experimental Procedures

### Bacterial strains, growth conditions, and helper phage

*Escherichia coli* strain TG1 (*supE thi-1* Δ[*lac-proAB*) Δ[*mcrB-hsdSM*]*5* [rK^−^ mK^−^] [F′ *traD36 proAB lacI*^q^*Z*Δ*M15*]) was used to construct and propagate all phagemid vectors and phage display library, and for obtaining stocks of the helper phage VCSM13 (Stratagene, La Jolla, California). TG1 containing the phagemid vectors or recombinant phagemids was infected with helper phage VCSM13 to allow packaging of phagemid DNA into phagemid particles (PPs) and display of *L. rhamnosus* proteins on the surface of the PPs. *Escherichia coli* cells were incubated in Yeast Extract Tryptone broth (2×YT) and *E. coli* transformants in 2×YT containing 20 μg/mL chloramphenicol (Cm) at 37°C with aeration. Lactobacilli were propagated in the Man–Rogosa–Sharpe (MRS) broth (Oxoid, Basingstoke, UK) at 37°C in liquid medium or on the Petri plates. Plates were incubated anaerobically. Bacteriological Agar (Oxoid) was used at 1% (w/v) for solid bacterial cultures. When required, antibiotics were added to media at the following concentrations: 20 μg/mL Cm and 100 μg/mL ampicillin (Amp) for *E. coli*; 5 μg/mL erythromycin (Em) for lactococci and lactobacilli; 5 μg/mL tetracycline (Tet) and 500 μg/mL streptomycin for lactobacilli. The detailed list of bacterial strains and their genotypes is given in Table S1.

### Phage infection, growth, purification, and titration

In the phagemid system used in this work, virions containing helper phage genomes are referred to as phage, whereas virions containing phagemid genomes are referred to as PPs. In all experiments in which phage or PPs were produced, an exponential-phase culture (around 10^8^ cells/mL) of the appropriate strain was infected with helper phage VCSM13 at a multiplicity of infection (m.o.i) of 50 (50 phage to 1 bacterium), for 30 min at 37°C. Infected cells were separated from unabsorbed helper phage by centrifugation at 5000*g* for 10 min at room temperature and resuspended in fresh medium. The cultures were then incubated for 4 h at 37°C with aeration unless otherwise stated. At the end of incubation, cells were chilled, titrated (as required), and then separated from the phage or PPs by centrifugation at 13,200*g* for 20 min at 4°C. Bacterial pellets were resuspended in the equal volume of the chilled medium supplemented with 7% (v/v) DMSO (dimethyl sulfoxide). Generally, 1-mL aliquots were taken, frozen on dry ice, stored at −80°C, and later used for the analysis of the proteins by SDS-PAGE (sodium dodecyl sulfate – polyacrylamide gel electrophoresis) and Western blotting.

The supernatant containing phages/PPs was centrifuged again and then passed through a 0.45-μm filter to eliminate any remaining bacterial cells. PPs were further purified and concentrated by precipitation in polyethylene glycol (PEG)/NaCl buffer (5% [w/v] PEG, 500 mmol/L NaCl) and resuspended in TN buffer (10 mmol/L Tris-HCl pH 7.6; 50 mmol/L NaCl) unless otherwise stated.

The number of infectious phage was determined by titration on TG1 strain. Approximate number of phage was first estimated by placing 10 μL drops of phage dilutions onto soft agar containing 0.2 mL of overnight culture of appropriate *E. coli* strain. Plaques in the area of absorbed drops were counted to determine approximate numbers of phage. For accurate titration of phage, dilution was adjusted to obtain 100–300 plaques per plate (based on the approximate titer). At least 300 plaques from the total of three plates were counted to determine the titer accurately. Helper phage titer was expressed as plaque-forming units per mL (pfu/mL).

The infectious PPs carrying a Cm^R^ marker were titrated by the same method, except that specially prepared double-layer plates were used, to allow in-agar infection of the indicator strain TG1 prior to exposure to Cm (Russel 1993). Briefly, 2×YT agar plates were prepared with 25 μg/mL chloramphenicol. These plates were overlaid with 9-mL chloramphenicol-free 2×YT agar shortly before use. PP stock was then mixed with *E. coli* strain TG1 in the soft agar layer as described earlier. PP titer was expressed as colony-forming units per mL (cfu/mL).

### Construction of the phagemid pYW01 carrying PelB signal sequence and colD origin of replication

The phagemid pYW01 was constructed in two steps: first, the *psp* (filamentous phage infection induced) promoter, ribosomal-binding site and *pelB* signal sequence (from *Erwinia carotovora* pectate lyase *–* PelB*)* were amplified by polymerase chain reaction (PCR) using pJARA144 (J. Rakonjac, unpubl. data) as a template and primers pDJ01F01, containing an EarI site, and pJARA144R02, containing an NsiI restriction site (Table S2). The PCR product was cleaved and ligated into the EarI-NsiI digested phagemid pDJ01 (Table S2). This step inserted the *pelB* signal sequence between the *psp* promoter and multiple cloning site cassette of pDJ01. The resulting phagemid was designated pDJ08. Next, the origin of replication in pDJ08 (*colEI*) was replaced with the *colD* origin of replication from the vector pGZ119EH (Table S2). This was achieved by cleavage of the *psp*-*pelB*-c-myc-*gIIIC* cassette and origin of replication from pDJ08 using PstI-ScaI followed by removal of 3′ protruding end from PstI by T4 DNA polymerase (Roche, Basel, Switzerland) and ligation of the resulting fragment to pGZ119EH EcoRV-ScaI fragment carrying *colD* origin of replication. The resulting phagemid pYW01 was used in the construction of *L. rhamnosus* HN001 shot-gun phage display library.

### Construction of the *L. rhamnosus* HN001 shot-gun phage display library

The library was constructed from mechanically (nebulization) sheared *L. rhamnosus* HN001 DNA and cloned into the phagemid vector pYW01. The fragments obtained varied in size between 0.3 and 4 kbp, with majority between 0.8 and 2 kbp. To eliminate fragments below 0.5 kbp, a protein concentrator with 100 kDa cutoff was used (Vivascience, Hannover, Germany).

Approximately 50 μg of the genomic fragments were ligated to 14 μg the SmaI-digested vector pYW01 using T4 DNA ligase (Roche). After phenol and chloroform extraction, the ligated DNA was ethanol precipitated, washed with 70% (v/v) ethanol, and dissolved in 70 μL H_2_O. *Escherichia coli* TG1 electrocompetent cells (Jacobsson et al. 2003) were transformed with 3 μL of ligation mix (2.5 kV, 25 μF, 400 Ω) in 2-mm-gap cuvettes. A total of 23 electrotransformations were carried out. The transformed cells were pooled, transferred to 230 mL of 2×YT, and incubated for 1 h at 37°C with rotatory agitation. After incubation, a 1 mL aliquot was taken to determine the number of transformants by plating on 2×YT agar with 20 μg/mL Cm. An aliquot (10 mL) of the remaining bacteria was transferred to 990 mL of 2×YT containing 20 μg/mL Cm and incubated at 37°C with aeration until exponential phase (OD_600_ ∼0.2). The exponentially growing culture was infected with wild-type helper phage VCSM13 (m.o.i = 50) for 30 min. Cells were then harvested by centrifugation at 3200*g* for 10 min and the pellet was resuspended in 1 L of 2×YT containing 20 μg/mL Cm. Infected cells were incubated for 4 h at 37°C with aeration. The host cells were pelleted by centrifugation and PPs were collected in the supernatant. These PPs represent the master phage display library. The library PPs were precipitated and purified as described in the phage manipulations section. They were stored in 7% (v/v) DMSO at −80°C.

### Affinity screening of the shot-gun phage display library for *L. rhamnosus* HN001 cells binders

*Lactobacillus rhamnosus* HN001-binding PPs were selected from the *L. rhamnosus* HN001 shot-gun library by affinity-enrichment protocol similar to the one described for the *Rhizobium leguminosarum* cell-surface-associated agglutinin (Ausmees et al. 2001) with slight modification. Briefly, strain HN001 to be used as bait was cultivated in MRS medium supplemented with 1 mmol/L CaCl_2_ at 37°C. The Ca^2+^ ions were added based on the evidence that these ions are essential in binding of some proteins to the cell surface via lectin domains (Armitage et al. 1992; Buts et al. 2003). Cells from 1.5 mL of the late exponential phase (∼1 × 10^9^ cells) were collected by centrifugation at 1100*g* for 5 min and the supernatant was carefully removed. The cell pellet was resuspended in 0.6 mL TBS (30 mM Tris, 150 mM NaCl, pH 7.6) containing 2 mmol/L CaCl_2_ and mixed with the *L. rhamnosus* HN001 phage display library (∼3 × 10^9^ PPs). Binding was allowed to proceed for 3 h at room temperature on a roller. Unbound PPs were removed by washing the cells five times with 1.5 mL TBS. After every wash, the bacteria were gently spun down at 1100*g* for 3 min in a microfuge. After the last wash, the cell pellet was resuspended in 0.05 mL 2×YT, transferred to a new tube and mixed with 0.5 mL of the exponentially growing *E. coli* TG1 culture to recover bound phage. This mixture was incubated for 30 min at 37°C to allow phage to infect the *E. coli* cells. Subsequently, concentrated infected *E. coli* TG1 cells (and dilutions thereof) were plated on selective double-layer chloramphenicol plates (described above) to, respectively, recover and titer the eluted PPs. Cm^R^ colonies were counted in diluted samples to determine the titer, whereas undiluted PPs were collected by scraping the plates with 2 mL 2×YT. An aliquot (0.5 mL) was used to inoculate 200 mL of 2×YT containing Cm. When the culture reached exponential phase, it was infected with wild-type helper phage VCSM13 in order to produce PPs of the enriched library. After the helper phage infection, the culture was processed, as described in the section on phage manipulation, to extract, concentrate, and purify PPs. These enriched PPs were then used in the subsequent round of panning according to the same protocol. Affinity-enrichment procedure was repeated three times (three rounds of panning were performed). After each round, the total number of eluted PPs was determined and profiles of plasmid dsDNA isolated from amplified library pools were monitored to estimate enrichment frequency. Distinct plasmid bands, enriched after the third cycle of panning, were purified from preparative agarose gel and used to transform the *E. coli* TG1 cells. Ten recombinants containing inserts of different sizes were further analyzed by DNA sequencing.

### Purification of PPs displaying SpcA and cell-binding assays

*Escherichia coli* clones containing the recombinant phagemid pSpcA^A^ (K2089), selected from the HN001 shot-gun library after panning on *L. rhamnosus* HN001 cells (encoding residues 1–376), and a clone containing recombinant phagemid pSpcA^S^ (K2090) identified as a secretome clone in the HN001 secretome library (encoding residues 1–199) (Jankovic et al. 2007) were used to generate PPs displaying corresponding fragments of protein SpcA. A volume of 100 mL of exponentially growing cultures of corresponding strains K2089 and K2090 was infected with helper phage VCSM13 at m.o.i of 50 phages per bacterium. The negative PP control was pYW01 PPs, generated by infection of strain K1978 (TG1//pYW01) as described above. Infected cells were incubated for 4 h at 37°C with aeration. The *E. coli* cells were pelleted by centrifugation and PPs were collected in the supernatant. The PPs were precipitated with PEG/NaCl, resuspended in 200 μL of TBS pH 7.4, and enumerated by titration. Binding of SpcA^A^-displaying particles to HN001 cells did not require Ca^2+^; hence, all binding assays with individual clones were done in the absence of CaCl_2_.

To determine binding of PPs displaying SpcA^A^, and SpcA^S^ fragments and binding of negative control containing empty vector pYW01, to HN001 and other tested strains, a binding assay that was equivalent to the panning protocol for library screening, described above, was used. The same protocol was used in binding assays to mutanolysin–lysozyme and proteinase-K-treated cells, except that the cells were treated with the appropriate enzymes and washed prior to assay, as described below.

For proteinase K treatment, HN001 cells from 1.5 mL of early stationary-phase culture were collected by centrifugation and washed once in TBS pH 7.4. The resulting cell pellet was then resuspended in 0.7 mL of buffer P(+) (100 mmol/L Tris-HCl pH 7.5, 5 mmol/L CaCl_2_ and 5 mg/mL proteinase K) and buffer P(−) (100 mmol/L Tris-HCl pH 7.5 and 5 mmol/L CaCl_2_), respectively. All samples were incubated 1 h at 37°C. Cells were then washed four times in the equal volume of cell-wash buffer (TBS pH 7.4, 1 mmol/L EDTA [ethylenediaminetetraacetic acid], protease inhibitor cocktail [Roche]), by pelleting and resuspension, followed by a 15-min incubation at 15°C for all washes. After the last wash, cells were resuspended in 0.6 mL of the same buffer and used immediately in the binding assay as described above for untreated cells. Buffer control for this experiment contained the cells treated in the same manner, except that proteinase K was omitted. To ensure that protease did not degrade pIII or displayed SpcA on the surface of PPs during binding assay (which would result in loss of infectivity and/or failure to bind HN001 cells due to degradation of pIII and SpcA, respectively), a mock protocol was set up using a mixture of cell-wash buffer (TBS pH 7.4, 1 mmol/L EDTA, protease inhibitor cocktail), and the proteinase-K-containing buffer, allowing for expected protease dilution in the PPs-cell-binding step. The titer remained the same, confirming no degradation of pIII. For lysozyme–mutanolysin (L-M) treatment, HN001 cells from 20 mL of late-exponential-phase culture were collected by centrifugation and resuspended in equal volume of prewarmed MRS broth. After the culture was incubated for another 2 h, cells from 1.5 mL of culture were collected and washed once in TBS pH 7.4. The cells were then resuspended in 0.5 mL of L-M buffer(+) (TBS pH 7.4, 25% [w/v] sucrose, 1 mmol/L EDTA, 20 mg/mL lysozyme, and 20 μg/mL mutanolysin) and L-M buffer(−) (TBS pH 7.4, 25% [w/v] sucrose and 1 mmol/L EDTA), respectively. After reaction was carried out at 37°C for 1 h, 0.5 mL of 0.25 mol/L EDTA was added. The mixture was incubated on ice for 5 min to stop the reaction. The resulting protoplasts were collected by gentle centrifugation (1000*g*), washed once with TBS, and resuspended in 0.6 mL of the assay buffer (TBS). Buffer control for this experiment contained the cells treated in the same manner, except that lysozyme and mutanolysin were omitted.

Binding efficiency in all cell-binding assays was determined as the ratio of total number of eluted PPs, to the total number of PPs used in the assay. The number of particles was determined by titration, as described in the phage manipulations section.

### Expression and purification of MBP-SpcA fusion protein

DNA sequence encoding the N-terminal portion of SpcA, containing both Big-3 domains, but lacking the signal sequence (SpcA^A1^, residues 117–309) was amplified by PCR, using genomic DNA as template and primers pWW37 and pWW50 (Table S3). The primers introduced EcoRI and PstI sites at the ends of the amplicon. The PCR product and the expression vector pMal-c2X (New England Biolabs, Ipswich, Massachusetts) were digested with EcoRI and PstI restriction enzymes and ligated to EcoRI-PstI-cut pMAL-c2X vector which contains maltose-binding protein (MBP) N-terminal tag to obtain recombinant plasmid pMBP-SpcA^A1^. The MBP-SpcA^A1^ fusion was then expressed in TG-1 cells and affinity purified using amylose resin (New England Biolabs) according to the manufacturer's instructions. Purity of the protein was analyzed by SDS-PAGE (Laemmli 1970).

### Screening of *L. rhamnosus* HN001 shot-gun phage display genomic library for gene(s) encoding SpcA-binding protein(s)

Purified MBP-SpcA^A1^ fusion was used as a bait to screen the HN001 shot-gun phage display library (described above) to identify SpcA-docking protein on the surface of HN001. Four rounds of panning were carried out. In the first round of panning, affinity binding to amylose resin (binds MBP tag of the MBP-SpcA^A1^ fusion with a high affinity; New England Biolabs) was used in the enrichment step of the library panning. Briefly, 4 μg MBP-SpcA^A1^ fusion was mixed with 10^11^ PPs from the library in a microcentrifuge tube. After incubation at room temperature for 3 h, 50 μL of amylose resin in TBS was added and the mixture was incubated for another hour. The amylose resin with bound proteins and phage particles was then collected by centrifugation and washed five times with TBS to eliminate unbound proteins and PPs. After the last wash, the bound PPs and MBP-SpcA^A1^ protein were eluted by resuspending the amylose resin in 500 μL of TBS containing 10 mmol/L maltose at room temperature for 15 min. Phage particles in complex with MBP-SpcA^A1^ fusion protein were amplified by infecting *E. coli* TG-1 to transduce the phagemid DNA; the transfected (Cm^R^) cells were then superinfected with VCSM13 helper phage to produce the PPs, which are concentrated and purified as described above for the library screening using HN001 cells as a bait. In the next three rounds of panning, the affinity-enrichment procedure was carried out in the microtiter plates (MaxiSorp™, Nunc, Rochester, New York). The bait protein (MBP-SpcA^A1^) and controls (MBP and bovine serum albumin [BSA]) were immobilized on the surface of microtiter plate wells at 4°C overnight, and the wells were subsequently blocked by 2% (w/v) BSA in TBS. About 3 × 10^9^ phage particles from the previous panning round on MBP-SpcA^A1^ bait were added. After incubation at and washing, MBP-SpcA^A1^-bound phage particles were eluted by 100 μL of low-pH elution buffer (100 mmol/L glycine-HCl, 0.1% [w/v] BSA, pH 2.2) at room temperature for 15 min and neutralized by 6 μL of 2 mol/L unbuffered Tris. Eluted PPs were amplified and concentrated, and the plasmid profiles after each round of panning were examined as described in the section of library screening for binding to HN001 cells. The profile of plasmids eluted from parallel controls, panned on BSA and MBP, was used as a comparison, to distinguish plasmid bands selected on the MBP-SpcA^A1^ fusion relative to those selected by panning on the controls. One plasmid band that was identified in the plasmid profile after the fourth round of panning on MBP-SpcA^A1^, but absent from the profiles of plasmids eluted from the same round of panning on negative controls (BSA and MBP), was excised from the gel and transformed into TG1. Inserts of ten individual transformants were analyzed by DNA sequencing to identify open readinf frames (ORFs) from which they are derived.

### Phage-based Western blot

To confirm binding of PPs displaying SpcB^N1^ and SpcB^N2^ fragments of protein SpcB to the SpcA^A1^, phage-Western blots (or PP-Western blots) were carried out. The MBP-SpcA^A1^ fusion protein was partially cleaved by the sequence-specific protease, Factor Xa (Novagen-Merck, Darmstadt, Germany), whose recognition site is between the MBP tag and the SpcA protein in pMBP-SpcA^A1^. The Factor Xa digestion reactions were prepared according to the product manual (New England Biolabs) and incubated at 37°C for 2 h. Two sets of digested proteins and undigested controls were analyzed by SDS-PAGE; gel portion containing one set of samples was stained by Coomassie brilliant blue and the portion containing a duplicate set was transferred to the nitrocellulose membrane for blotting with SpcB^N1^- and SpcB^N2^-displaying PPs. The membranes were blocked in TBST (TBS; 0.5% [v/v] Tween-20) containing 5% (w/v) skim milk at 4°C overnight. The membranes were subsequently exposed to 1 × 10^9^ phage particles in TBST at room temperature for 2 h and the unbound phage particles were washed off with TBS. The bound phage particles were in turn detected by primary antibodies against M13 phage coat protein pVIII (Sigma-Aldrich, St. Louis, Missouri) and alkaline phosphatase-conjugated secondary antibodies using the standard Western blot protocols. The PP-SpcB^N1^ and PP-SpcB^N2^ – bound bands on the membrane – were detected by alkaline phosphatase assay (Sambrook and Russell 2001).

## Results

### Construction of a new phage display vector, pYW01, and a shot-gun phage display library of *L. rhamnosus* HN001

**Figure 1:**
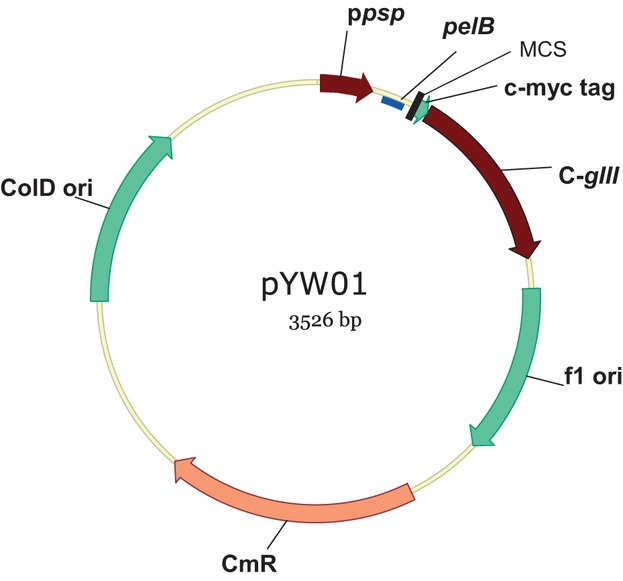
Phagemid vector used in construction of *Lactobacillus rhamnosus* HN001 shot-gun phage display library. Vector features (in the clockwise direction) are p*psp*, phage shock protein promoter; *pelB*, encoding a PelB protein signal sequence; MCS, multiple cloning cassette; c-myc tag, a common peptide tag; *C-gIII*, C-domain of *gIII*; f1 ori, the f1 phage origin of replication for generation of ssDNA for packaging into the phagemid particles; CmR, chloramphenicol resistance cassette; colD ori, the colD plasmid origin of replication.

A phage display phagemid vector suitable for bacterial shot-gun phage display libraries was constructed, to avoid toxicity of bacterial proteins to *E. coli* and improve display efficiency. The display cassette is expressed from a phage-shock-protein (*psp*) promoter, induced by the helper filamentous phage infection. The *psp* promoter is only expressed after the helper phage infection and was shown to minimize toxicity of pIII fusions that is often encountered in phage display (Beekwilder et al. 1999). The display cassette of this vector (named pYW01) contains the PelB signal sequence, a multiple cloning site, followed by a c-myc tag fused to the C-terminal domain of pIII (pIIIC; Fig. 1). The truncated pIII (pIIIC) in pYW01 lacks the N-terminal domains of pIII which cause resistance to filamentous phage infection when expressed in host *E. coli* cells (Rakonjac et al. 2011). This is important in construction of bacterial genomic shot-gun phage display libraries, to avoid resistance to infection with the helper phage (required for production of PPs and display of proteins encoded by the inserts) when the inserts contain bacterial promoters upstream of the ORFs encoding displayed proteins.

A shot-gun phage display library of HN001 was constructed in this new vector, as described in Experimental Procedures. The inserts of the library ranged from 0.3 to 4 Kbp; the primary size of the library was 3 × 10^8^ clones. The library was first amplified using the plasmid origin of replication (in the absence of a helper phage). In the next step, the amplified library was mass infected with the *gIII*^+^ helper phage VCSM13 to initiate replication from the phagemid f1 origin and packaging into PPs. These PPs were expected to display the whole HN001 proteome and contain corresponding DNA sequences inside the virions. PPs released from the infected library were collected, purified by PEG precipitation, and used in affinity screenings.

### SpcA is a cell-binding protein of *L. rhamnosus* HN001

Cell-surface-binding proteins in bacteria may be involved in a number of functions, including cell aggregation, or they can be surface-associated enzymes or adhesins. We undertook to functionally select for cell-surface-binding proteins by affinity screening of an HN001 phage display library for recombinant PPs that bind the HN001 cells.

For the enrichment procedure, strain HN001 cells were used as bait to enrich for cell-adhering phagemid clones from the library. The library enrichment for PPs that bind to HN001 cells was monitored by increase in the number of eluted PPs after each progressive round of panning. A 916-fold increase in the number of eluted PPs between rounds one and three indicated that specific enrichment had taken place. Plasmid profiles of enriched library pools after each round of panning were monitored by agarose gel electrophoresis as another indicator of enrichment for selected clones (Data S1 and Fig. S1). Before the panning, the random size distribution of inserts resulted in a smear upon agarose electrophoresis of purified plasmids, whereas after the second and third round discrete plasmid bands were observed, indicating enrichment for plasmids of specific sizes. The most prominent three bands seen on the agarose gel after the third panning round were each excised, purified, and used to separately transform *E. coli* host strain TG1. Sequence analyses of inserts from ten transformants revealed that all contained the 5′-moiety of a single ORF of a secretome protein, in frame with the c-myc tag and *gIIIC*. The ORF sequence was preceded by a putative ribosomal-binding site and initiated by an ATG start codon. Upstream of the transcriptional site a -10 box (ACATAAAAT) and -35 box (TTGATT) was identified in the putative promoter region using BPROM promoter prediction program from the Softberry server (http://www.softberry.com/berry.phtml). Translated products of the inserts encoding the selected ORF ranged in size from 334 to 376 amino acids. Searching of an *L. rhamnosus* HN001 genome sequence identified this ORF as *lrh*_07061 and its product is annotated as a cell-surface protein precursor (accession number ZP_03210506 within the contig NZ_ABWJ01000002.1). As the first *L. rhamnosus* protein whose cell-binding properties are experimentally proven, the locus and the gene were renamed *spcA* (surface protein, cell-binder A).

The signal sequence prediction algorithm SignalP 4.0 (Petersen et al. 2011) revealed that the *spcA* encodes an extracellular protein with an amino-terminal signal sequence. Furthermore, Pfam, CDART, CDD, and SMART database searches (Schultz et al. 1998; Geer et al. 2002; Finn et al. 2006; Marchler-Bauer et al. 2011) detected possible functional domains, two bacterial immunoglobulin-like type 3 domains (Big-3; E-values 4e^−04^ and 3e^−05^). The Ig-like domains are commonly found in the surface and secreted proteins in both prokaryotes and eukaryotes and are typically involved in extracellular adhesion and binding processes (Hultgren et al. 1993; Takai and Ono 2001; Fraser et al. 2007).

**Figure 2:**
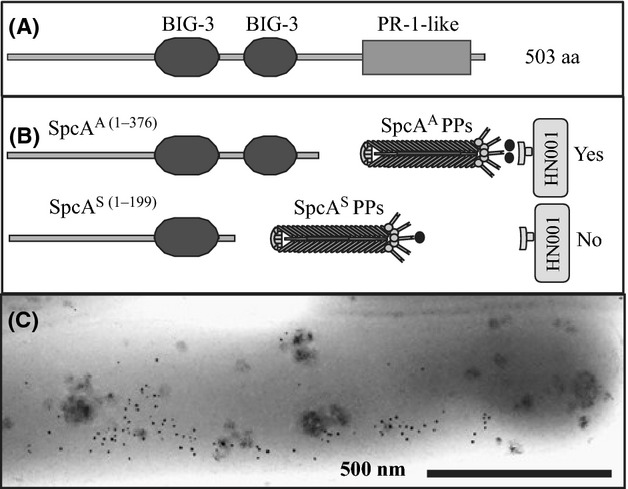
SpcA protein domain organization and mapping of HN001-binding domains. (A) SpcA protein domain organization. SpcA protein consists of two bacterial immunoglobulin-like domains family 3 (Big-3) and a putative PR-1-like domain. (B) Mapping of HN001-binding domains. The phagemid particles (PPs) displaying both Big-3 domains (SpcA^A^ PPs) were capable of binding to HN001 cells, whereas the phage particles displaying only N-terminal Big-3 domain (SpcA^S^ PPs) lost the binding capacity. The numbers in parentheses indicate the length of displayed fragment (in amino acid residues). (C) Electron micrograph of gold-labeled SpcA^A^ PPs attached to the surface of an HN001 cell.

The translated complete ORF *of spcA* was 503 residues in length (1512 bp). In addition to the Big-3 domains, SpcA in all sequenced *L. rhamnosus* strains contains a third domain that is C-terminal to the Big-3 domains. This domain was identified as an SCP-like extracellular protein domain (SCP_bacterial; CD05379; E = 4e^−3^). The SCP protein was initially identified as a murine extracellular glycoprotein (Mizuki and Kasahara 1992), and the SCP-like domains are found in diverse extracellular proteins of bacteria and eukaryotes. Some members of this family are endopeptidases, while some are transglycosylases or have nonenzymatic roles. Analysis using the PHYRE protein structure prediction web server (Kelley and Sternberg 2009) selected a member of this family from plants, pathogen-recognition protein PR-1-like, as the top structural homolog along the whole length of this domain; therefore, this domain in SpcA was denoted as PR-1-like (Fig. 2A). The equivalent ORF to SpcA in the genome sequence of *L. rhamnosus* GG is denoted as LGG_01989 (Kankainen et al. 2009). Interestingly, although the SpcA amino acid sequence over 503 residues is 100% and 97% conserved with those of *L. rhamnosus* strains GG and Lc 705, respectively, in the two latter strains this ORF has additional 68 residues at the C-terminus, whereas the HN001 ORF is disrupted by an IS5-like element.

A secretome database/clone bank, a resource of displayed purified surface and secreted proteins from *L. rhamnosus* HN001 constructed previously in our laboratory (Jankovic et al. 2007), contained a clone that displayed a truncated spcA (ACC61714) that encoded a 199-residue fragment. This fragment was shorter than inserts selected for binding to HN001 cells; it encoded only one (N-terminal) Big-3 domain. To distinguish between the shorter SpcA insert from the clone bank, and a longer affinity-selected larger insert, the shorter one was named SpcA^S^ (for short), whereas the longer one was named SpcA^A^ (for affinity selected). The short fragment, which encoded only the N-terminal Big-3 domain, was used for mapping of the HN001-binding portion of SpcA. PPs displaying this N-terminal 199-residue long SpcA^S^ fragment showed no binding above the vector background (Fig. 2). Therefore, for interaction with the HN001 surface structures, sequence downstream of the N-terminal Big-3 domain, containing the C-terminal Ig-like domain, is required. Binding of the SpcA^A^ to the cells was confirmed by transmission electron microscopy which detected gold-labeled SpcA^A^-displaying PPs bound to the surface of HN001 cells (Fig. 2C).

When a BLASTP search (Altschul et al. 1997) was performed with the complete amino acid sequences from HN001 SpcA or its GG homolog, a hit with low conservation to the second (C-terminal) Big-3 domain of SpcA (E = 5e^−3^) but with a highly conserved PR-1-like domain and downstream 68-residue extension found in GG but not in HN001 (E = 6e^−67^ to HN001 and E = 1e^−85^ to GG) was identified (*Lactobacillus zeae* KCTC 3804; accession number ZP_09454360.1). Other hits had a relatively low significance; the homologies were limited to the pair of Big-3 domains and were not secreted, but are rather LPXTG cell-wall-anchored proteins found in genera *Lactobacillus*, *Enterococcus*, and *Listeria*.

Together, these observations indicated that the *spcA* ORF encodes a *L. rhamnosus* – unique secreted protein with a propensity for multiple interactions through two Big-3 domains and one PR-1-like domain.

### SpcA binds to a cell-wall-associated protein unique to *L. rhamnosus* species

**Figure 3:**
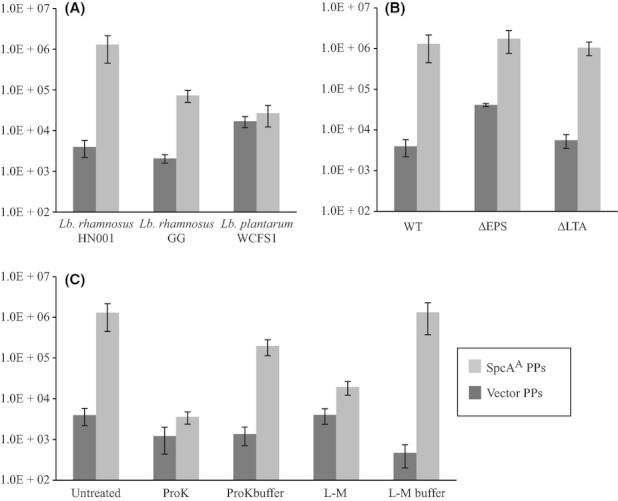
Lrh33 cell-binding assays. In these assays, binding of SpcA^A^-displaying phagemid particles (PPs) that were selected from HN001 phage display library (SpcA^A^ PPs; Fig. 2) was compared with binding of the negative control PPs derived from empty vector pYW01 (Vector PPs) as described in Experimental Procedures. (A) Binding assays with different *Lactobacillus* strains and species. (B) Binding assays with the wild-type (WT) *L. rhamnosus* HN001 and mutants; ΔEPS, mutant lacking an extracellular exopolysaccharide; ΔLTA, mutant with an altered lipoteichoic acid. (C) Binding assays with lysozyme–mutanolysin (L-M) treated HN001 and proteinase K treated HN001 cells. Untreated, HN001 cells in phosphate buffer saline (PBS); ProK, proteinase-K-treated cells; ProK buffer, cells processed in the same manner as in the ProK assay, except that the proteinase K was omitted; L-M, lysozyme–mutanolysin-treated cells (spheroplasts); L-M buffer, cells processed as L-M cells, except that the lysozyme and mutanolysin are omitted.

To determine whether SpcA has a role in cell–cell aggregation, a deletion of *spcA* gene was constructed by marker exchange, as described in Figure S2. The *ΔspcA* mutant did not demonstrate any difference from the wild type in aggregation (not shown), suggesting that this is not an aggregation protein. Given that the SCP-like or PR-1-like domains in other organisms are versatile and have been reported to perform a variety of functions, from enzymatic to host-defense, we hypothesized that the SpcA Big-3 domains may bind to the bacterial cell in order to immobilize the PR-1-like domain on the surface of the cell. In that case, it is expected that SpcA would be anchored through a cognate species-specific docking molecule. In order to determine the SpcA tropism or species specificity, we tested the binding profile of SpcA^A^-displaying PPs (SpcA^A^ PPs) to various lactobacilli. SpcA^A^ showed binding to *L. rhamnosus* GG, but not to different lactobacillus species, *Lactobacillus plantarum* strains WCFS1 (Fig. 3A) and ATCC14197, *Lactobacillus sakei* Lb790 and *Lactobacillus acidophilus* NCFM (data not shown). Therefore, Lrh33 binds to a ligand that is most likely species specific for *L. rhamnosus*.

Next, we sought to determine the nature of the SpcA-docking molecule on the surface of *L. rhamnosus* cells. We tested binding of SpcA^A^ PPs to the mutant HN001 lacking an extracellular polysaccharide or with an altered lipoteichoic acid (LTA), but these mutations did not affect binding of SpcA^A^ PPs (Fig. 3B). To test whether SpcA^A^ PPs interact with the surface proteins, their binding to the proteinase-K-treated cells was tested (Fig. 3C). Proteinase K eliminated binding of SpcA^A^ PPs. Furthermore, SpcA^A^ PPs did not bind to spheroplasts, which were obtained by L-M treatment of HN001. These enzymes removed the cell wall, together with the cell-wall-anchored proteins, from the surface of the cells. In summary, lack of binding to protease-treated cells and spheroplasts and binding to cells lacking an exopolysaccharide (EPS) or with an altered LTA suggests binding to a cell-wall-anchored protein.

### SpcB, the largest surface protein in *L. rhamnosus* genome, is the SpcA-docking protein

**Figure 4:**
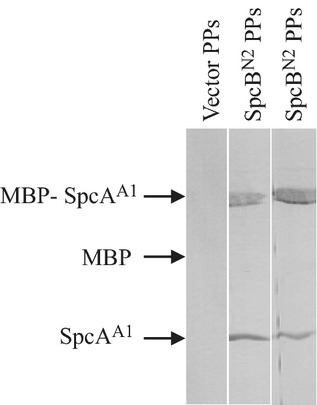
Confirmation of the physical interaction between SpcA and SpcB by phagemid particles (PPs) Western blot. Affinity-purified MBP-SpcA^A1^ was first partially cleaved by a protease, Factor Xa, which cuts between the MBP and SpcA^A1^. Uncleaved fusion protein and proteolysis products, MBP and SpcA^A1^, were separated by SDS-PAGE and transferred to nitrocellulose membranes. The membranes were cut along the lanes in the gel, and each strip was blotted with one of the three PP samples: Vector pYW01 PPs, PPs containing empty vector (negative control); SpcB^N1^ PPs, PPs displaying SpcB^N1^ (residues 59–391); SpcB^N2^ PPs, PPs displaying SpcB^N2^ (residues 211–595); MBP, maltose-binding protein N-terminal tag. The bound PPs were detected using primary rabbit anti-M13 phage antibodies, followed by anti-rabbit IgG secondary antibodies conjugated with alkaline phosphatase, and visualized by alkaline phosphatase enzymatic assay.

To identify SpcA ligand(s), we undertook another affinity screening of the HN001 phage display library, and this time using as bait purified MBP fusion to the SpcA^A^ fragment that lacks signal sequence (residues 117-309; named MBP-SpcA^A1^). The first round of panning was carried out by immobilizing the MBP-SpcA^A1^, in complex with associated library PPs, on the amylose resin which captures the MBP tag fused to SpcA^A1^ fragment. The following three rounds of panning were performed using MBP-SpcA^A1^ fusion immobilized on the microtiter plate. In the rounds 2–4, the library was first depleted from MBP-binding clones by first binding to immobilized MBP tag; the remaining unbound PPs were then used for binding the MBP-SpcA^A1^ fusion. Washing and elution steps were performed for both the MBP and MBP-SpcA^A1^ and plasmid profiles were analyzed for both eluates, to distinguish between SpcA^A1^- and potential MBP-binding PPs. Analysis of the plasmid profiles after the fourth round of panning indicated enrichment of discrete plasmid bands. The bands that were present in the eluate from the immobilized MBP-SpcA^A1^ but absent from the MBP control eluate (Fig. S3) were excised from agarose gels and transformed into *E. coli* TG1 strain to obtain individual clones. Ten transformants were analyzed by DNA sequencing; the inserts in five clones encoded the N-terminal domain of SpcB, the largest protein in the HN001 genome (3275 residues in length). This protein is encoded by *spcB* gene, located in the same cluster of *L. rhamnosus*-unique genes with *spcA*. These five transformants represent two clones containing distinct but overlapping inserts, named SpcB^N1^ and SpcB^N2^, encoding amino acid residues 32–391 and 211–586, respectively. Both inserts were in frame with upstream vector-encoded PelB signal sequence and downstream c-myc tag and C-terminal domain of pIII, confirming that they were able to be displayed on the surface of PPs. To confirm binding to SpcA^A1^, rather than the MBP portion of the bait, the MBP-SpcA^A1^ fusion was digested with the sequence-specific protease Factor Xa, whose cleavage site is engineered between the MBP and SpcA^A1^. Binding of SpcB^N1^- and SpcB^N2^-displaying PPs to either of the proteolytic products was determined by a phage-Western blot. This is a blot in which the partially digested MBP-SpcA^A1^ fusion, separated by SDS-PAGE and transferred onto a nitrocellulose membrane, is probed with the SpcB^N1/2^-displaying PPs (and the empty vector control PPs), followed by detection of bound phage using antiphage antibodies. This experiment showed specific binding of the SpcB^N1^ PPs and SpcB^N2^ PPs to SpcA^A1^ and to the uncut MBP-SpcA^A1^ fusion, but not to the MBP band, proving that this is specific SpcA^A1^–SpcB^N^ interaction (Fig. 4). The sequence overlap between the two inserts, SpcB^N1^ and SpcB^N2^, determines an SpcA^A1^-binding domain, from residue 211–391.

**Figure 5:**
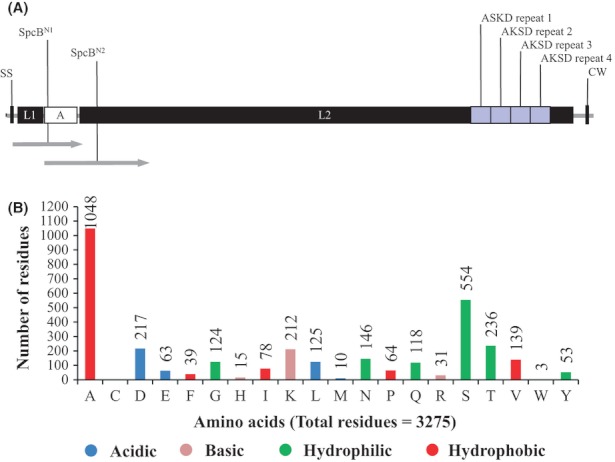
SpcB protein organization and mapping of SpcA-binding region. (A) Schematic representation of the SpcB organization. SS, the KxYKxGKxW signal sequence; L1, low-complexity region I; A, high-complexity region containing SpcA-binding sequence; L2, low-complexity region II; CW, cell-wall anchor, including LPXTG motif. The gray block arrows represent the two N-terminal SpcB fragments that were affinity selected from the HN001 phage display library using purified MBP-SpcA^A1^ protein as bait. (B) Plot of the SpcB amino acid composition.

The SpcB primary sequence organization (Fig. 5) resembles that of large glycosylated Ser-rich surface adhesins of Gram-positive bacteria, including the characteristic signal sequence that contains KxYKxGKxW motif within an extended N-terminal positively charged sequence, and a glycine-rich hydrophobic region (Bensing et al. 2007). The high-complexity SpcA-ligand-binding domain (A region) is preceded by a relatively short Ala-Ser-Thr-rich low-complexity region (L1; residues 67–213) at the N-terminus of the mature protein and is followed by a nearly 3000-residue long low-complexity Ala-Ser-rich domain (L2; residues 389-3178) in *L. rhamnosus* HN001 and GG (Kankainen et al. 2009; Morita et al. 2009). As a difference from other Gram-positive Ser-rich proteins, SpcB does not have a short highly conserved Ser-rich motif; instead, it is organized as imperfect repeats of about 100 residues in length. Four almost identical 111-residue repeats are located between residues 2591 and 3034; these repeats are more hydrophobic than the upstream low-complexity region. Besides the predominant Ala (44) and Ser (20) residues, these repeats contain a large number of charged residues, mainly Lys (14) and Asp (13), with other residues having low representation and were therefore named ASKD repeats. Relatively high frequency of these four residues is maintained along the whole low-complexity region which makes up most of the protein (Fig. 5B). Along the whole length of the low-complexity region, the PHYRE server predicts both the disordered structure (with high probability) and α-helical secondary structure (with variable probability). It is therefore possible that this part of the molecule forms a helical fibril. It is noteworthy that the SpcB sequence between residues 401 and 1209, when fused to a signal-sequence-less pIII, was able to target the fusion to the *E. coli* membrane (Jankovic et al. 2007; Jankovic 2009); therefore, this region of SpcB is likely to be able to insert into lipid bilayers. The C-terminal LPXTG motif (Schneewind et al. 1992) and transmembrane helix of SpcB correspond to a typical cell wall anchor. Finding that the ligand is a *L. rhamnosus*-unique cell-wall-anchored protein is consistent with the SpcA^A^ PP binding assays described in the previous section. In GG, as in HN001, SpcB is 3275 residues in length, whereas in Lc 705 it has 3390 residues, due to an extra 111-residue repeat within the Ala-Ser-rich region, and several other short insertions/duplications.

Whereas no homologs were identified for the Ala-Ser-rich sequence which represents the majority of the protein (when the BLASTP search was carried out with the low-complexity filter turned on), the high-complexity SpcA-binding domain had low similarity to one protein in the NCBI database, a hypothetical KxYKxGKxW-signal sequence protein of the human gut bacterium *Lactobacillus parafarraginis* F0439 (ZP_09393738.1). This hypothetic protein has 32% identity (BLASTP; E = 3e^−12^) to SpcB between residues 233 and 449, corresponding roughly to the N-terminal high-complexity domain that mediates binding to SpcA Big3 domains; furthermore, the two proteins have a conserved signal sequence (residues 1–60; 57% identity; E = 2e^−10^ by BLAST analysis). The SpcA-binding domain is not annotated in the pfam database and did not give significant hits in the PHYRE three-dimensional (3D) alignment, and hence, it may be considered a unique domain. The remaining portion of *L. parafarraginis* ZP_09393738.1 protein also has a low-complexity Ala-Ser-rich region arranged in imperfect repeats.

## Discussion

We report a discovery of two extracellular proteins that appear to form a complex on the surface of the probiotic bacterium *L. rhamnosus* HN001 This complex was identified by affinity screenings of an *L. rhamnosus* HN001 phage display library. In the first screen, the cell-binding protein SpcA was identified using the *L. rhamnosus* HN001 cells as bait. Purified SpcA was, in turn, used to identify its ligand (or docking protein), SpcB, a large cell-wall-anchored adhesin/fibril-like protein. Both proteins are unique to *L. rhamnosus*, with only a distant homolog of docking protein found in the genome of *L. parafarraginis*. These findings are consistent with the species specificity in cell-binding assays using SpcA-displaying PPs.

Mapping of binding domains determined that the two bacterial immunoglobulin-like domains 3 (Big-3) of SpcA interacted with an N-terminal high-complexity domain of SpcB. SpcA also contains a domain that showed the best match in a 3D alignment with the bacterial PR-1-like domain, but also aligns weakly with β-glycanases.

**Figure 6:**
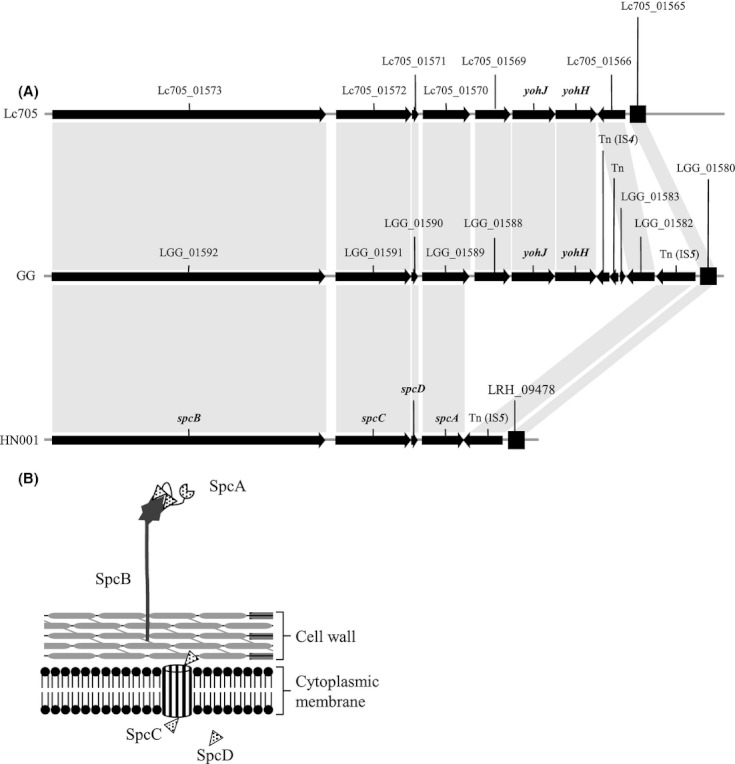
(A) Organization of *spcB–spcA* gene cluster in *Lactobacillus rhamnosus* strains Lc 705, GG, and HN001 and GG. (B) Model of the SpcB–SpcA complex and position of SpcC and SpcD.

The SpcA-docking protein SpcB has a signal sequence motif and organization typical of Gram-positive glycosylated adhesins, exemplified by the GspB adhesin of *Streptococcus gordonii* that binds to human platelets, and a mucus-binding fibril-like adhesin of *Streptococcus parasanguinis*, Fap1 (Wu et al. 1998; Bensing et al. 2007). Even though the signal sequence of SpcB has characteristics typical of the glycosylated Ser-rich adhesins, the gene organization surrounding the *spcB* ORF is very different from that of *gspB* and *fap1*. The *gspB* and *fap1* genes are in the same cluster/operon with two glycosydase-encoding genes and genes encoding for a secondary/alternative Sec translocon complex (SecA2, SecY2 and in some cases SecE2 and SecG2) (Rigel and Braunstein 2008; Zhou and Wu, 2009). The secondary translocon, either in combination with or independently of the standard translocon (SecA, SecYEG), is required for export of GspB and Fap1 from their respective organisms. The same gene cluster also typically encodes two or more transmembrane or cytosolic accessory proteins that either facilitate or are required for the secretion of glycosylated precursors (Rigel and Braunstein 2008; Zhou and Wu 2009). In contrast, the *spcB–spcA* cluster in HN001 includes only two other genes: *spcC*, encoding a transmembrane protein containing two Big-3 domains; and *spcD*, encoding a small cytosolic protein containing a single Big-3 domain (Fig. 6A). Analysis of sequence downstream sequence of ORF *spcA* in HN001 revealed the presence of an ORF, transcribed in the opposite direction, encoding a putative transposase within a predicted transposable element, possibly belonging to insertional element of IS5 family (IS*5*). Using the FindTerm bacterial terminators prediction algorithm (http://www.softberry.ru/berry.phtml), two terminator sequences were detected: one upstream of putative transcriptional start of *spcB* and one bordering the putative IS5 element. No terminator sequences were detected using the same prediction software between the *spcB*, *spcC, spcD,* and *spcA* ORFs (Jankovic 2009).

This cluster of ORFs (*spcB–spcA*) is conserved in the GG genome (LGG_01592–LGG_01589) and the genomes of other *L. rhamnosus* strains (Kankainen et al. 2009; Morita et al. 2009; Yu et al. 2011; Pittet et al. 2012; Prajapati et al. 2012). However, starting from the stop codon after the residue 503 of *spcA*, the HN001 sequence contains an IS*5*-like element, whereas GG (ATCC 53103), Lc 705, and ATCC 8530 contain the complete *spcA* ORF, followed by two additional ORFs that encode two predicted glycosyltransferases (GtfA family) that are normally found in the same gene cluster as large KxYKxGKxW-signal-sequence-containing adhesins and are required for glycosylation of Fap1 adhesin (Zhou and Wu 2009). Downstream of *spcA* are three IS elements in GG, but none in Lc 705. Interestingly, the *spcA*-inserted IS5-like element of HN001 is at the end of the NZ_ABWJ01000002.1 contig; to determine the sequence beyond the end of this contig, we used a series of primers corresponding to *spcA*-downstream sequence in GG. This analysis detected a deletion that removed seven ORFs between *spcA* and the IS*5* element in HN001 that corresponds to the second IS element in GG, downstream from the *spcA* (Fig. 6).

Even though HN001 lacks the two GtfA family glycosyltransferases, it contains four additional GtfA homologs that are highly conserved to both missing glycosyltransferases. Therefore, it is possible that SpcB is still glycosylated in HN001, despite the deletion. Whatever possible differences in modification between HN001 and GG, they do not affect binding of SpcA, as shown in Figure 3.

The alternative Sec system has not been annotated in any of the sequenced *L. rhamnosus* genomes. Given that *L. rhamnosus* has only a single complement of genes annotated as *secA* and *secYEG*, it is expected that SpcB is exported *via* the standard SecA-SecYEG system. Proteins encoded by the two ORFs between SpcB and SpcA could be accessory proteins involved in secretion of glycosylated SpcB.

The reported proteomic analysis of *L. rhamnosus* GG surface proteins identified, using mass spectroscopy, 105 different peptides from SpcB (LGG_01592) (Savijoki et al. 2011). This protein is therefore produced in *L. rhamnosus* GG and targeted to the cell wall. The mass fingerprinting approach used in Savijoki et al. (2011) cannot assign peptides to a protein if they are glycosylated; therefore, many proteolytic peptides derived SpcB in *L. rhamnosus* GG must be unmodified (Lebeer et al. 2012).

The Ala-Ser-rich region of SpcB has some characteristics of α-helical membrane-inserting bacterial proteins. The PHYRE server predicted helical secondary structure along this region, but at the same time predicted, with high probability, disordered sequence. Given that we demonstrated that the Ala-Ser-rich region of SpcB (residues 401–1209) can insert into phospholipid bilayers (Jankovic et al. 2007), this portion of the molecule could target *L. rhamnosus* to phospholipid bilayers, including membranes of host epithelial cells. Alternatively, depending on the extent of glycosylation and the number of copies on the surface of *L. rhamnosus* cells, SpcB could regulate hydrophobicity or hydration of the cell surface.

Using a phage display approach, we identified a protein complex, representing a putative fibril-like-docking protein SpcB and attached soluble protein, SpcA, displaying a PR-1-like domain (Fig. 6B). SpcB–SpcA complex, due to the PR-1-like domain of SpcA and membrane-penetrating hydrophobic region of SpcB, has potential to mediate specific protein–protein interactions, membrane-insertion, or cell-surface-hydrophobicity regulation functions, all of which are pertinent to interaction with the environment and host.

## Supporting Information

**Figure S1.** The plasmid profiles over three rounds of affinity-screening (panning) of the HN001 phage display library using HN001 as bait. Lanes: pYW01, replicative form (covalently closed circular double-stranded DNA; cccDNA) of the phagemid vector pYW01 used to construct the phage display library; Round I; Round II; Round III; purified pools of the recombinant library (cccDNA) after indicated rounds of panning. Three black arrows indicate the DNA bands of enriched phagemids.

**Figure S2.** Construction of the HN001 Δ*spcA* mutant. (A) Vector used for construction of double cross-over mutant. (B) Map of the *spcA* deletion mutant and the wild-type *spcA* locus in HN001. (C) Confirmation Southern blotting using upstream and downstream probes (indicated in B).

**Figure S3.** The plasmid profiles over four rounds of affinity-screening (panning) of the HN001 phage display library using purified MBP-SpcA fusion protein as bait. The panning was carried out over four rounds of affinity selection and amplification (I–IV) to enrich the specific binders. From the second to the fourth rounds of panning, MBP and BSA were also included as control baits. pYW01 represents the phagemid used to construct the phage display library.

**Table S1.** Bacterial strains.

**Table S2.** Plasmids and phage.

**Table S3.** Oligonucleotides.

**Data S1.** Experimental procedures.
