# Supplementary material for: Unique secreted–surface protein complex of Lactobacillus rhamnosus, identified by phage display
Source: Microbiologyopen. 2012 Dec 11;2(1):1–17. doi: 10.1002/mbo3.53 (PMC3584209; doi:10.1002/mbo3.53)
Supplement: Supplementary file 1 [file mbo30002-0001-SD1.pdf]

Supporting Information

Table S1. Bacterial strains

Strain	*Genotype/comment*	*Reference*
Escherichia coli TG1	*supE thi-1* Δ(*lac-proAB*) Δ(*mcrBhsdSM*)*5* ($rK^{-}mK^{-}$) [F′ *traD36 proABlacI*^{q}*Z*Δ*M15*]	Zymo
E. coli K1978	TG1//pDJ08	This study
E. coli K1981	TG1//pYW01	This study
E. coli K2089	TG1//pSpcA^{S}	This study
E. coli K2090	TG1//pSpcA^{A}	This study
E. coli K2208	TG1//pMBP-SpcA^{A1}	This study
E. coli K2232	TG1//pSpcB^{N1}	This study
E. coli K2233	TG1//pSpcB^{N2}	This study
Lactococcus lactis MG1363	NCDO (prophage-cured)	(Gasson, 1983)
Lactobacillus rhamnosus HN001	Wild-type	(Prasad et al., 1998)
Lb. rhamnosus L002	HN001 *rpsL*(Sm^{r})	Fonterra Research and Development Centre (FRDC)
Lb. rhamnosus L024	HN001 *rpsL*(Sm^{r}) *ΔspcA*::Tet^{r}	This study
Lb. rhamnosus L003	HN001 *welE*::Em^{r} (insertion mutant)	FRDC
Lb. rhamnosus L006	HN001 *rpsL*(Sm^{r}) *ΔdltD::*Tet^{r} (marker replacement mutant)	FRDC
Lb. rhamnosus GG	ATCC53103; Wild-type	(Kankainen et al., 2009; Morita et al., 2009)
Lb. plantarum WCFS1	Wild type	(Kleerebezem et al., 2003)

Table S2. Plasmids and phage

Plasmids	***Description***	***Resistance***	***Reference***
pAK100	Phage display vector	Chloramphenicol	(Krebber et al., 1996)
pJARA144	pPSP2 *Δrom* (high copy number variant)	Ampicillin	(Beekwilder et al., 1999); J. Rakonjac, unpublished
pDJ01	Bacterial secretome-selective phage display vector containing ColE1 origin of replication	Chloramphenicol	(Jankovic et al., 2007)
pDJ08	pDJ01 containing signal sequence	Chloramphenicol	This work
pGZ119EH	Cloning vector containing ColD origin of replication	Chloramphenicol	(Lessl et al., 1992)
pYW01	Phage display vector containing ColD1 origin of replication	Chloramphenicol	This work
pSpcA^{S}	pDJ01::SpcA (1-199)	Chloramphenicol	This work
pSpcA^{A}	pYW01::SpcA (1-376)	Chloramphenicol	This work
pMal-c2X	Expression vector for construction of N-terminal MBP fusions	Ampicillin	New England Biolabs
pMBP-SpcA^{A1}	pMBP-c2X::SpcA(117-309)	Ampicillin	This work
pFRC027	A shuttle vector for marker-replacement mutagenesis in *Lb. rhamnosus* HN001	Tetracycline Erythromycin RpsL wild-type (Streptomycin sensitive)	FRDC unpublished
pSpcANKO	pFRC027-derived knock-out construct for deletion of *spcA* (Fig. S2)	Tetracycline Erythromycin RpsL wild-type (Streptomycin sensitive)	This work

Table S3. Oligonucleotides

Name	*Sequence*	*Restriction site*	*Application*
pDJ01F03	ATGTTGCTGTTGATTCTTCA		sequencing
pDJ01R02	CCGGAAACGTCACCAATGAA		sequencing
pDJ01F01	CCGCCGAATCCCGGAAGAGCTGCAGCATGATGAAATTC	EarI	cloning
pJARA144R02	GCCGCCATGCATGGGCCATGGCCAT	NsiI	cloning
pWW37	CCGGAATTCAATAACCCAGAGCCGGAAGC	EcoRI	cloning
pWW50	AAAACTGCAGCTATTATTTTGGATCCATAAAACTAAAACG	PstI	cloning
pWW18	AAGGAAAAAAGCGGCCGCGCTGCCGGTGTCCTCAAAGG	NotI	cloning
pWW19	CAATTCCCATGGAGATCTCTCGAGCCATACCCTCCAAACTTCAGTG	NcoI	cloning
pWW20	AATGCCCTAGGGTCGACCCCGGGTTTTACTGATGCCCCAACCGG	SalI	cloning
pWW21	GGACTAGTGGACGGTAAAAACTCACGCCC	SpeI	cloning

Supplementary Figures

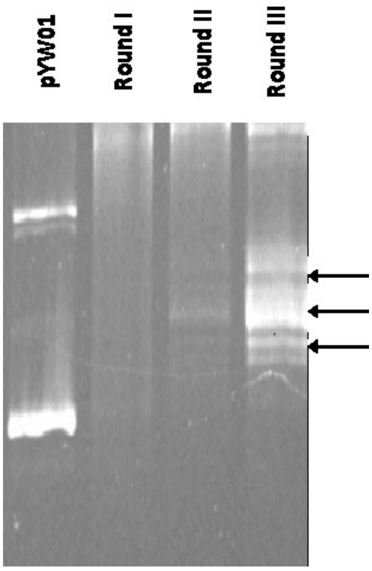

Figure S1. The plasmid profiles over three rounds of affinity-screening (panning) of the HN001 phage display library using HN001 as bait. Lanes: pYW01, replicative form (covalently closed circular double-stranded DNA; cccDNA) of the phagemid vector pYW01 used to construct the phage display library; Round I; Round II; Round III; purified pools of the recombinant library (cccDNA) after indicated rounds of panning. Three black arrows indicate the DNA bands of enriched phagemids.

.

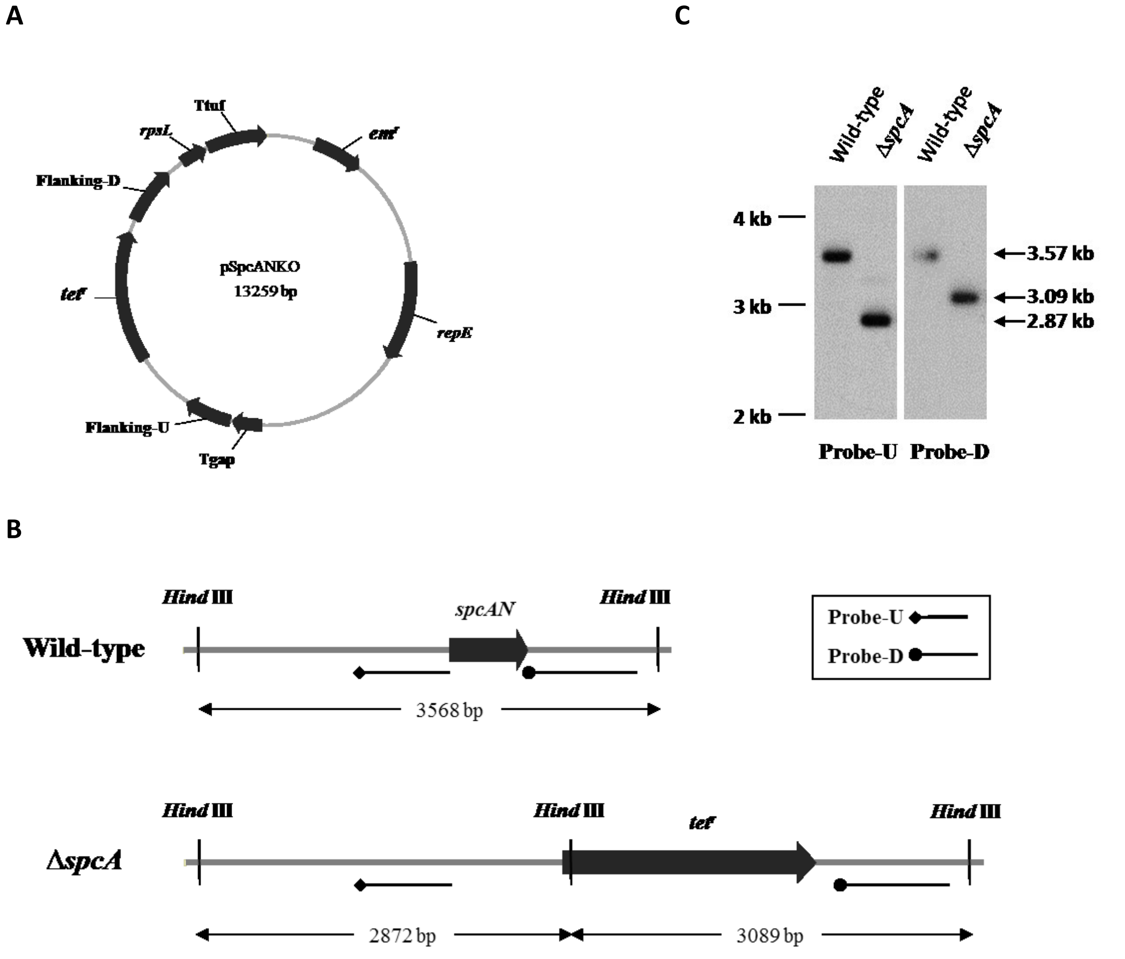

Figure S2. Construction of the HN001 Δ*spcA* mutant. A) Vector used for construction of double cross-over mutant. **B)** Map of the *spcA* deletion mutant and the wild-type *spcA* locus in HN001. **C)** Confirmation Southern blotting using upstream and downstream probes (indicated in B).

The deletion mutant was constructed by marker replacement (double-crossover), in which a (Tet^r) marker replaced the 5' moiety (*spcAN*; ~0.6 kbp) of *spcA* gene *via* homologous recombination. This deletion eliminates production of SpcA due to deletion of its promoter and the lack of translational start downstream of Tet^r-encoding gene. The 3' end of *spcA* (3' 180 nucleotides) and the downstream DNA had not been sequenced at the time when the knock-out mutant was made, however the subsequent genome sequencing of HN001 showed that no CDS follows *spcA* in the same transcriptional direction, and that an IS5-like sequence, which is present in the genome in multiple copies, is inserted into the 5' end of spcA gene in such orientation that the transposase gene is transcribed from the complementary strand and therefore in the opposite orientation relative to *spcA*. Hence, no polar effect on

other genes was possible in this mutant. Briefly, two homologous fragments flanking the 5' end of *spcA* were amplified by PCR using tailed primers that included appropriate restriction sites (Supporting information, Table S3) and chromosomal HN001 DNA as a template. The two PCR products, upstream and downstream, cleaved with NotI-NcoI and SalI-SpeI, respectively, were sequentially inserted into the *Lactobacillus rhamnosus* HN001-specific replacement vector pFRC027 NotI-NcoI and SalI-SpeI sites flanking the Tet^r marker, to obtain the SpcA replacement recombinant plasmid pSpcANKO (A). Vector pFRC027 and its derivatives cannot be maintained in *E. coli*, therefore construction of the pSPCANKO had to be carried out in *Lactococcus lactis* laboratory strain MG1363. The recombinant plasmid was confirmed by DNA sequencing. In addition to Tet^r marker, the vector pFRC027 also has an erythromycin-resistance (Em^r) marker and a wild-type *rpsL* gene from *Lb. gasseri* DSM20243 that confers dominant streptomycin-sensitive (Sm^s) phenotype to transformed HN001 *rpsL* (Sm^r) host, and which allows positive selection of double cross-over of the Tet^r marker flanked by inserted sequences which is accompanied by the loss of the plasmid backbone. The recombinant plasmid was electroporated into *L. rhamnosus* HN001 *rpsL* (Sm^r), a spontaneous streptomycin-resistant mutant of HN001. The electro-competent cells were prepared and electroporated according to a previously reported protocol (Varmanen et al., 1998). The erythromycin resistant transformants of HN001 *rpsL* (Sm^r) strain were selected and the colonies were used to inoculate MRS broth containing both Tet and Sm, to select for Tet^r and Sm^S double-crossover recombinants in which, the Tet^r-encoding gene replaced the *spcA* sequence (B), whereas the plasmid backbone carrying dominant wild-type *rpsL* (Sm^s) gene was lost. Bacteria were further replica-plated on Em-containing MRS agar plates to confirm the loss of Em^r marker and hence the loss of the plasmid backbone. Em^s clones were analysed by diagnostic PCR and Southern blotting (C) to confirm the correct genetic replacement.

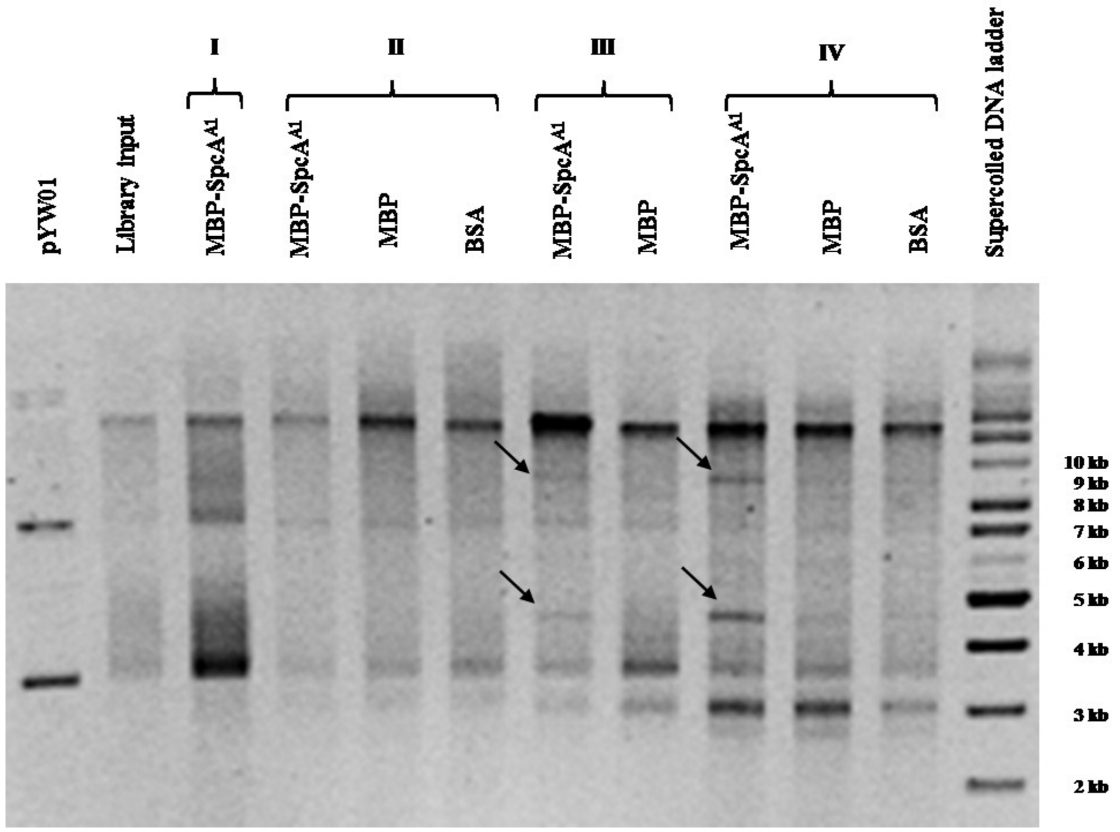

Figure S3. The plasmid profiles over four rounds of affinity-screening (panning) of the HN001 phage display library using purified MBP-SpcA fusion protein as bait. The panning was carried out over four rounds of affinity selection and amplification (I to IV) to enrich the specific binders. From the second to the fourth rounds of panning, MBP and BSA were also included as control baits. pYW01 represents the phagemid used to construct the phage display library.

Supplementary Experimental Procedures

Genomic DNA purification from* L. rhamnosus *HN001

Chromosomal DNA was isolated from *L. rhamnosus* HN001 based on a slightly modified protocol (Prasad et al., 1998). 20 ml of stationary HN001 culture was prepared by adding 1% (v/v) inoculum (frozen stationary culture) to 20 ml of MRS broth and incubating at 37 °C anaerobically for about 18 h. Cells were obtained by centrifugation at 6000 x g at room temperature for 10 min and resuspended in 20 ml of pre-warmed MRS broth. The culture was then incubated at 37 °C in a water bath for another two hours. After incubation, cells were pelleted by centrifugation at 6000 x g for 10 min and washed twice with 4 ml of Buffer A (50 mM NaCl, 30 mM Tris, pH 8.0, 5 mM EDTA). Cell pellet was then resuspended in 0.5 ml of Buffer B [25% sucrose, 50 mM Tris pH 8.0, 1mM EDTA, Lysozyme (20 mg ml^{-1}), Mutanolysin (20 µg ml^{-1})]. This reaction was carried out at 37 °C for 1 h. To stop the reaction, 0.5 ml of 0.25 M EDTA pH8.0 was added and the mixture was incubated at room temperature for 5 min. After that, 200 µl of 20% (w/v) SDS was added to lyse the cells and the mixture was incubated at 65 °C for up to 90 min until the solution became relatively clear. To degrade proteins, 20 µl of proteinase K (20 mg ml^{-1} in H_2O) was added and the reaction was incubated at 65 °C for 15 min. Then equal volume of phenol/chloroform (1:1) solution was added to the cell lysate and mixed thoroughly. After incubation at room temperature for 5 minutes, the mixture was centrifuged at 4000 x g for 10 min. The aqueous phase containing DNA was collected carefully to minimise the protein contamination at the interface. This phenol/chloroform extraction was repeated for another 2 - 3 times until protein at interface was minimal. To eliminate RNA contamination, DNase-free RNase (Roche) was added up to final concentration of 100 µg ml^{-1} and the mixture was incubated at 37 °C for 30 min. After that, phenol/chloroform extraction was performed 3 - 4 times and aqueous phase containing chromosomal DNA was collected. To precipitate chromosomal DNA, two volumes of cold 95% (v/v) ethanol were added and the mixture was incubated at 4 °C overnight. DNA pellet was then obtained by centrifugation at 14100 x g for 20 min, washed with 70% (v/v) cold ethanol and air dried at room temperature. Finally, DNA pellet was resuspended and dissolved in 10 mM Tris-HCl pH 8.0 at 4 °C overnight.

References

Beekwilder, J., Rakonjac, J., Jongsma, M., and Bosch, D. (1999). A phagemid vector using the E-coli phage shock promoter facilitates phage display of toxic proteins. *Gene* **228**, 23-31.

Gasson, M.J. (1983). Plasmid complements of Streptococcus lactis NCDO 712 and other lactic streptococci after protoplast-induced curing. *J Bacteriol* **154**, 1-9.

Jankovic, D., Collett, M.A., Lubbers, M.W., and Rakonjac, J. (2007). Direct selection and phage display of a Gram-positive secretome. *Genome Biol* **8**, R266.

Kankainen, M., Paulin, L., Tynkkynen, S., von Ossowski, I., Reunanen, J., Partanen, P., Satokari, R., Vesterlund, S., Hendrickx, A.P., Lebeer, S.*, et al.* (2009). Comparative genomic analysis of Lactobacillus rhamnosus GG reveals pili containing a human-mucus binding protein. *Proc Natl Acad Sci U S A* **106**, 17193-17198.

Kleerebezem, M., Boekhorst, J., van Kranenburg, R., Molenaar, D., Kuipers, O.P., Leer, R., Tarchini, R., Peters, S.A., Sandbrink, H.M., Fiers, M.W.*, et al.* (2003). Complete genome sequence of Lactobacillus plantarum WCFS1. *Proc Natl Acad Sci U S A* **100**, 1990-1995.

Krebber, A., Burmester, J., and Pluckthun, A. (1996). Inclusion of an upstream transcriptional terminator in phage display vectors abolishes background expression of toxic fusions with coat protein g3p. *Gene* **178**, 71-74.

Lessl, M., Balzer, D., Lurz, R., and Waters, V.L. (1992). Dissection of IncP conjugative plasmid transfer: definition of the transfer region Tra2 by mobilization of the Tra1 region *in trans*. *J Bacteriol* **174**, 2493-2500.

Morita, H., Toh, H., Oshima, K., Murakami, M., Taylor, T.D., Igimi, S., and Hattori, M. (2009). Complete genome sequence of the probiotic Lactobacillus rhamnosus ATCC 53103. *J Bacteriol* **191**, 7630-7631.

Prasad, J., Gill, H., Smart, J., and Gopal, P. (1998). Selection and characterisation of Lactobacillus and Bifidobacterium strains for use as probiotics. 8: 993-1002. *Int Dairy J* **8**, 993-1002.

Varmanen, P., Rantanen, T., Palva, A., and Tynkkynen, S. (1998). Cloning and characterization of a prolinase gene (pepR) from *Lactobacillus rhamnosus*. *Appl Environ Microbiol* **64**, 1831-1836.
